# Impact of DNA Extraction Method on Variation in Human and Built Environment Microbial Community and Functional Profiles Assessed by Shotgun Metagenomics Sequencing

**DOI:** 10.3389/fmicb.2020.00953

**Published:** 2020-05-25

**Authors:** Hui-yu Sui, Ana A. Weil, Edwin Nuwagira, Firdausi Qadri, Edward T. Ryan, Melissa P. Mezzari, Wanda Phipatanakul, Peggy S. Lai

**Affiliations:** ^1^Division of Pulmonary and Critical Care Medicine, Massachusetts General Hospital, Boston, MA, United States; ^2^Division of Infectious Diseases, Massachusetts General Hospital, Boston, MA, United States; ^3^Harvard Medical School, Boston, MA, United States; ^4^Mbarara University of Science and Technology, Mbarara, Uganda; ^5^Infectious Diseases Division, International Centre for Diarrhoeal Disease Research, Bangladesh, Dhaka, Bangladesh; ^6^Alkek Center for Metagenomics and Microbiome Research, Department of Molecular Virology and Microbiology, Baylor College of Medicine, Houston, TX, United States; ^7^Division of Immunology, Boston Children’s Hospital, Boston, MA, United States

**Keywords:** microbiome, microbiota, shotgun metagenomics sequencing, DNA extraction method, built environment, human microbiome

## Abstract

Both the host microbiome and the microbiome of the built environment can have profound impacts on human health. While prior studies have suggested that the variability introduced by DNA extraction method is less than typical biologic variation, most studies have focused on 16S rRNA amplicon sequencing or on high biomass fecal samples. Shotgun metagenomic sequencing provides advantages over amplicon sequencing for surveying the microbiome, but is a challenge to perform in lower microbial biomass samples with high human DNA content such as sputum or vacuumed dust. Here we systematically evaluate the impact of four different extraction methods (phenol:choloroform, and three high-throughput kit-based approaches, the Promega Maxwell gDNA, Qiagen MagAttract PowerSoil DNA, and ZymoBIOMICS 96 MagBead). We report the variation in microbial community structure and predicted microbial function assessed by shotgun metagenomics sequencing in human stool, sputum, and vacuumed dust obtained from ongoing cohort studies or clinical trials. The same beadbeating protocol was used for all samples to focus our evaluation on the impact of kit chemistries on sequencing results. DNA yield was overall highest in the phenol:choloroform and Promega approaches. Only the phenol:choloroform approach showed evidence of contamination in negative controls. Bias was evaluated using mock community controls, and was noted across all extraction methods, although Promega exhibited the least amount of bias. The extraction method did not impact the proportion of human reads, although stool had the lowest proportion of human reads (0.1%) as compared to dust (44.1%) and sputum (80%). We calculated Bray-Curtis dissimilarity and Aitchison distances to evaluate the impact of extraction method on microbial community structure by sample type. Extraction method had the lowest impact in stool (extraction method responsible for 3.0–3.9% of the variability), the most impact in vacuumed dust (12–16% of the variability) and intermediate values for sputum (9.2–12% variability). Similar differences were noted when evaluating microbial community function. Our results will inform investigators planning microbiome studies using diverse sample types in large clinical studies. A consistent DNA extraction approach across all sample types is recommended, particularly with lower microbial biomass samples that are more heavily influenced by extraction method.

## Introduction

There is currently an explosion in the number of scientific papers exploring the role of the microbiome in human health. The link between the human microbiome and diseases has spanned all organ systems, with studies linking the gut microbiome to diseases from inflammatory bowel disease (Lloyd-Price et al., 2019) to neurologic disorders such as Parkinson’s disease (Sampson et al., 2016). Human mucosal surfaces previously thought to be largely sterile, such as the lung, are now known to possesses a distinct microbiome. Lung microbial communities have been associated with specific lung diseases (Sze et al., 2012) and may have causal links to disease progression (O’Dwyer et al., 2019). In parallel, there is increasing recognition that the microbiome of the environment, in particular the built environment, can impact the development (Kirjavainen et al., 2019) or severity (Lai et al., 2018) of asthma. Technological advances in isolating DNA from human samples and decreasing costs of sequencing have made it possible to study microbial communities in these low-biomass sample types. However, factors that influence or bias results of low-biomass (or samples of variable biomass) sequencing have not been well-studied, leading to uncertainty about the best study design for these sample types in microbiome studies.

Sample collection, preservation, homogenization, storage, DNA extraction method, DNA fragment library preparation, sequencing platform, and the bioinformatics pipelines used to process sequencing data are factors known to introduce variation in microbiome study results (Sinha et al., 2017). The technical factor with the greatest impact on variability is DNA extraction method (Costea et al., 2017), though one recent study suggests that the variability introduced by any technical factor (including DNA extraction method) is less than the natural biological variation in stool samples (Sinha et al., 2017). Another study reported that in other sample types, such as saliva, DNA extraction methods including both phenol:chloroform and kit-based approaches have no impact on microbial community assessment (Lim et al., 2017). A major limitation of existing studies evaluating the impact of DNA extraction methods is the inclusion of largely high biomass samples such as stool (Claassen et al., 2013; Wagner Mackenzie et al., 2015; Gerasimidis et al., 2016; Janabi et al., 2016; Costea et al., 2017) and/or a focus on amplicon rather than shotgun metagenomics sequencing (Marotz et al., 2017). Studies comparing DNA extraction methods in different sample types for shotgun sequencing are lacking. Shotgun sequencing provides advantages over amplicon sequencing including species or strain-level taxonomic resolution and the ability to infer microbial community function, and is less influenced by amplification bias (Brooks et al., 2015). However, shotgun metagenomics sequencing has other unique challenges including higher input DNA requirements, the presence of “contaminant” host reads that must be removed prior to analysis (in effect decreasing sequencing depth), and higher cost.

Our primary goal was to determine the impact of DNA extraction method on variability in microbiome profiles assessed by shotgun metagenomics sequencing in diverse sample types of variable biomass: human stool, human induced sputum, and vacuumed dust. These sample types were chosen because the vast majority of microbiome studies are performed in fecal samples. Therefore, stool serves as the typical high biomass human sample, sputum represents a low biomass human sample, and vacuumed dust a low biomass environmental sample. We used archived samples from existing cohort studies or clinical trials from both low- and high-income countries dating as far back as 2001 to improve the generalizability of our results for researchers planning to leverage their own existing richly phenotyped cohorts for microbiome applications. We selected the phenol:chloroform approach as the comparator group given studies suggesting that DNA yield is highest with this approach (Janabi et al., 2016), and three popular commercial kits that use a magnetic bead-based approach, the Promega Maxwell gDNA Kit (referred to as Promega), Qiagen MagAttract PowerSoil DNA Kit (referred to as Qiagen), and ZymoBIOMICS 96 MagBead Kit (referred to as Zymo). Magnetic bead-based approaches have demonstrated higher DNA recovery than spin column-based approaches (Moeller et al., 2014; Dunbar et al., 2018), and with the use of automated extraction instruments, reduces labor costs and facilitates processing of high sample volume for large studies. We used the same beadbeating protocol across all methods to focus on the impact of kit chemistries. We evaluated the performance of each approach using metrics that have been previously shown to be impacted by extraction method including DNA yield, proportion of human reads, evidence of contamination in negative controls, bias in positive controls using mock communities, and impact on resulting microbial communities in terms of species identified and predicted microbial community function.

## Materials and Methods

### Subject Recruitment and Sample Collection

**FIGURE 1:**
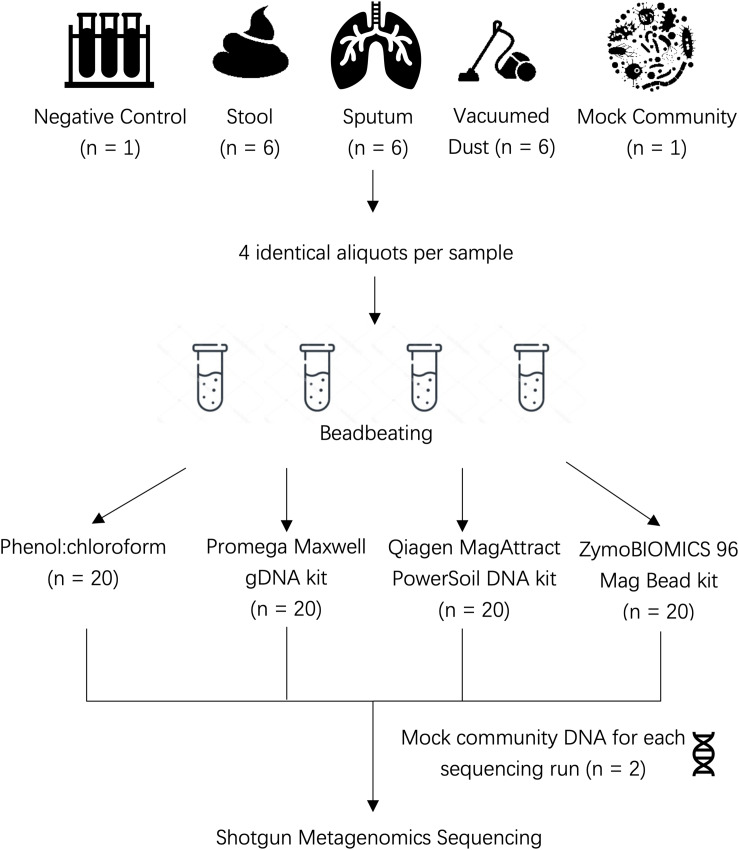
Overview of study design.

Human stool, sputum, and vacuumed dust were obtained from existing cohort studies or clinical trials as described below (see Figure 1 for overview of study design).

#### Sample Type 1: Human Stool

De-identified stool samples were obtained from a cohort study of culture-confirmed cholera cases and their household contacts (Weil et al., 2009) enrolled at the International Centre for Diarrhoeal Disease Research, Bangladesh (icddr,b) cholera treatment center in Dhaka, Bangladesh between September 23, 2001 and April 26, 2016. Stool samples from six patients were included in this study; two collected in 2001, two collected in 2009, and two collected in 2016. Stool samples were placed in cryovials and frozen at −80°C until transport on dry ice from Dhaka, Bangladesh, to Boston, Massachusetts, United States where they were placed in −80°C for long term storage. Collection and secondary use of de-identified stool specimens was approved by the Ethical Review Committee of the icddr,b (FWA00001468) and the institutional review board of Massachusetts General Hospital (Protocol # 1999P009116). Participants or their guardians provided written informed consent.

#### Sample Type 2: Human Induced Sputum

Induced sputum was obtained from participants recruited for a randomized controlled trial of small-scale chicken farms in rural Uganda (ClinicalTrials.gov NCT02619227) (Kakuhikire et al., 2016). Samples were obtained between 2015 and 2017. Prior to sputum induction, each participant rinsed their mouth with a 0.12% chlorhexidine gluconate mouthwash for 60 s to decrease oral contamination. Using a sterile disposable nebulizer setup, 3% saline (or subsequently 7% saline if the participant was not able to cough) was nebulized for up to 20 min. Under trained nursing supervision, participants were coached to expectorate into a sterile specimen cup. Sputum samples were visually assessed for quality prior to freezing at −80°C until transport on dry ice from Mbarara, Uganda to Boston, Massachusetts, United States where they were stored at −80°C. Prior to DNA extraction, six de-identified sputum samples were thawed, manually homogenized using a sterilely cut pipette tip, then aliquoted. The study was approved by the Research Ethics Committee of Mbarara University of Science and Technology (Protocol # 30/11-14), the Ugandan National Council of Science and Technology, with permission from the President’s Office in Uganda. This study was approved by the institutional review board of Massachusetts General Hospital (Protocol # 2015P000227). Participants provided written informed consent.

#### Sample Type 3: Vacuumed Dust

Vacuumed dust was obtained from the School Inner City Asthma Intervention Study (SICAS-2, ClinicalTrials.gov Identifier: NCT02291302), an ongoing randomized controlled trial to determine whether a school and classroom-based environmental intervention would reduce indoor air pollution and improve asthma morbidity (Phipatanakul et al., 2017). Children from schools in a northeastern United States city were recruited. Vacuumed dust was obtained during the school year using an Oreck XL vacuum (model BB870-AD; Oreck, LLC) fitted on the inlet hose with a dust collector filter (DUSTREAM^®^ collector, Indoor Biotechnology) according to a standardized protocol (Mitchell et al., 1997). After sample collection, dust was frozen at −80°C. Six dust samples from different classrooms collected between 2015 and 2016 were included in this study. This study was approved by the Institutional Review Boards of Boston Children’s Hospital (Protocol # IRB-P00006413) and Massachusetts General Hospital (Protocol # 2019P003489). Participating children provided assent and their guardians written informed consent. Permission for environmental sampling was obtained from the participating school systems.

#### “Positive” Mock Community Controls

To assess for bias in extraction and/or sequencing, commercially available mock community controls were used. These mock communities are composed of eight bacteria (three Gram-negative and five Gram-positive with a range of GC content) and two yeasts, with the following relative abundance: 12% *Pseudomonas aeruginosa*, 12% *Escherichia coli*, 12% *Salmonella enterica*, 12% *Lactobacillus fermentum*, 12% *Enterococcus faecalis*, 12% *Staphylococcus aureus*, 12% *Listeria monocytogenes*, 12% *Bacillus subtilis*, 12% *Saccharomyces cerevisiae*, 12% *Cryptococcus neoformans*. We used two types of these controls; (1) Mock community microbes (ZymoBIOMICS Catalog #D6300) where we pipetted 75 μL aliquots into four Lysing Matrix E tubes; and (2) Mock community DNA obtained by pooling DNA extracted from pure cultures (ZymoBIOMICS Catalog #D6306); this was used as the input DNA for library prep in each of the two sequencing runs for this study. The same lot number for each mock community type was used in this study.

#### Negative Reagent-Only Controls

For each extraction method, we included a negative reagent-only control where an empty Lysing Matrix E tube was used to assess for contamination present in extraction reagents or as a result of the extraction protocol. The position of the negative and positive controls relative to study samples for each extraction method in a 96-well plate was determined using a random number generator. In addition, for each sequencing run, the elution buffer (either DEPC-Treated, DNase/RNase free molecular biology grade water or 25 mM Tris-HCl) were included as samples for library prep.

### DNA Extraction

#### Beadbeating Protocol

We used the same beadbeating protocol for all sample types in order to isolate the impact of kit chemistries (buffers and reagents) on our results. A total of six stool samples, six sputum samples, and six dust samples were included in this comparison (Figure 1). Four replicates of each sample were placed into individual Lysing Matrix E tubes (MP Biomedicals) in the following amounts prior to DNA extraction: 100 mg of stool, 500 μL of sputum, and 200 mg of vacuumed dust. Lysing matrix E tubes were chosen as they contain 1.4 ceramic spheres, 0.1 mm silica spheres and one 4 mm glass bead; this range of bead sizes allows efficient lysis of diverse human and environmental sample types. Each replicate was then processed using each of the four extraction methods. To each tube, 750 μL of lysis buffer was added as follows: Cetyl Trimethyl Ammonium Bromide (CTAB) for Methods 1 and 2 below, PowerBead Solution/RNase A Solution for Method 3 below, ZymoBIOMICS Lysis Solution for Method 4 below. Beadbeating occurred for a total of 6 cycles at 7.0 m/s with each cycle lasting 30 s followed by 90 s pause. Pauses were incorporated to avoid overheating the sample due to friction generated by beadbeating. Sample lysate was then used for downstream extraction according to each protocol.

#### Magnetic Bead-Based DNA Extraction Using the KingFisher Flex Instrument for Methods 2–4

The KingFisher Flex benchtop automated extraction instrument (Thermo Fisher Scientific, Waltham) was used for high-throughput DNA extraction for Methods 2–4 as described below. In comparisons of different automatic extraction instruments, the Kingfisher platform has previously been shown to have superior yield for sequencing applications (Marotz et al., 2017). In this approach, the lysate after beadbeating for each sample is placed in 96-well plates with magnetic beads used to transfer samples between plates for binding, washing, and elution steps. Our comparison methods focused on magnetic bead-based extraction protocols due to the need for a high-throughput extraction pipeline and prior literature suggesting that DNA yields are improved with magnetic bead-based protocols compared to spin column-based protocols (Moeller et al., 2014; Dunbar et al., 2018).

#### Method 1: Phenol:Chloroform

Microbial DNA extraction was performed using a modified cetyltrimethylammonium bromide–polyethylene glycol (CTAB) phenol:chloroform extraction protocol used in urban asthma studies focused on the built environment microbiome as previously described (Fujimura et al., 2014; Lai et al., 2018). Briefly, this protocol involves the addition of CTAB for sample lysis followed by a heating step at 65°C for 15 min, addition of phenol:chloroform:isoamyl alcohol (25:24:1), beadbeating, then transfer of the supernatant to heavy phase-lock gel tubes (5Prime). One volume of chloroform is then added to each sample, which is centrifuged briefly. Linear acrylamide is added to the supernatant followed by a 2-h incubation with PEG at room temperature, washing with ice-cold 70%, air drying, and resuspension in molecular-grade H_2_O.

#### Method 2: Promega Maxwell HT 96 gDNA Blood Isolation System (Catalog #A2671)

Microbial DNA extraction was performed based on Technical Manual #TM473 available on www.promega.com/protocols. Modifications were made to the protocol for use with the KingFisher Flex rather than the Maxwell^®^ RSC instrument with kit chemistries similar to the Maxwell RSC PureFood GMO and Authentication Kit. Briefly, CTAB is added to sample aliquoted in beadbeating tubes, heated to 95°C for 5 min followed by beadbeating, addition of proteinase K, incubation at 70°C for 10 min, centrifuged, then the sample lysate transferred to 96-well plates for subsequent binding, washing, and elution steps on the KingFisher Flex. The kit binding buffer was replaced with 100% isopropanol in this protocol.

#### Method 3: Qiagen MagAttract PowerSoil DNA KF Kit (Catalog #27000-4-KF)

Microbial DNA extraction was performed using the Earth Microbiome Protocol (Marotz et al., 2017) with a modification to the beadbeating step as described above. A step-by-step description of this protocol is outlined in https://www.protocols.io/view/earth-microbiome-project-emp-high-throughput-htp-d-pdmdi46. Briefly, PowerBead Solution, RNase A, and SL Solution (a lysis butter) is added to each beadbeating tube followed by a heating step at 65°C for 10 min, beadbeating, transfer of the supernatant to 96-well plates for subsequent binding, washing, and elution steps on the KingFisher Flex.

#### Method 4: ZymoBIOMICS 96 Magbead DNA Kit (Catalog #D4302)

Microbial DNA extraction was performed according to manufacturer instructions, with the exception of the beadbeating step as described above. This kit has similar chemistries to other ZymoBIOMICS kits including the Miniprep kit, Microprep kit, and 96 DNA kit, with an adaptation made for automated magnetic bead platforms. Briefly, lysis solution and proteinase K was added to each sample in beadbeating tubes, the sample was incubated at 55°C for 30 min, followed by beadbeating, transfer of the sample lysate to 96-well plates for subsequent binding, washing, and elution steps on the KingFisher Flex.

### DNA Quantification, Library Prep, and Metagenomic Sequencing

Extracted DNA samples were quantified by the Quant-iT PicoGreen dsDNA Assay (Life Technologies), a fluorescent nucleic acid stain specific for double-stranded DNA (dsDNA). DNA was normalized to a concentration of 50 pg/μL. Illumina sequencing libraries were prepared from 100–250 pg of DNA using the Nextera XT DNA Library Preparation kit (Illumina) according to the manufacturer’s recommended protocol. Prior to sequencing, libraries were pooled by collecting equal volumes (200 nl) of each library from batches of 96 samples. Insert sizes and concentrations for each pooled library were determined using an Agilent Bioanalyzer DNA 1000 kit (Agilent Technologies). Metagenomic libraries were randomized to be sequenced on two individual lanes on the HiSeq platform (Illumina), targeting ∼2.5 Gb of sequence per sample with 150 base pair, paired-end reads.

Library prep and sequencing was performed at the Alkek Center for Metagenomics and Microbiome Research, Department of Molecular Virology and Microbiology, Baylor College of Medicine. Samples that failed library preparation (library fragment size below 470 bp and final library yield below 1.4 nM) were excluded from sequencing on this basis.

### Read-Level Quality Control, Assessment of Human “Contaminant” Reads, and Metagenomic Profiling

Sequencing reads were derived from raw BCL files which were retrieved from the sequencer and called into fastqs by Casava v1.8.3 (Illumina). Raw fastq sequences underwent quality trimming and Illumina adapter removal using bbduk (BBMap version 37.58, Bushnell, 2014). Trimming parameters were set to a kmer length of 17, allowing one mismatch, a min Phred quality score of 20 and entropy value of 0.7. Reads with a minimum average quality score below 20 and length shorter than 50 bp after trimming were discarded. The trimmed fastqs were then mapped to a hg38 reference database [Genome Reference Consortium Human Build 38 patch release 13 (GRCh38.p13), PRJNA31257] using bowtie2 version 2.3.5.1 (Langmead and Salzberg, 2012), with end-to-end and very-sensitive parameters in order to remove host contamination reads. A custom in-house script was used to remove the host reads from the trimmed fastqs for further taxonomic and functional profiling.

Taxonomic profiling of the sequenced samples was done using MetaPhlAn2 version 2.6.0 (Segata et al., 2012). Processed fastq reads were first mapped against the MetaPhlAn2 marker gene database (mpa_v20_m200) using bowtie2 (version 2.3.5.1) with the end-to-end, very-sensitive parameters. Each sample was run through the metaphlan.py script to generate the kingdom-specific taxonomic profile per sample, using the flag to generate relative abundances and estimated read counts. A custom in-house script was employed to merge the output for all samples into a single sample per taxon table for each kingdom and relative abundance and estimated read count output. Finally, the tables were converted into biom-format for further statistical analysis.

Functional profiling of the microbial community was done using HUMAnN2 version 0.11.1 (Franzosa et al., 2018). The standard recommended workflow was followed with bowtie2 and diamond version 0.9.15 (Buchfink et al., 2015) for the nucleotide and translated alignment steps, respectively. This created the pathway abundance and coverage tables, as well as gene family abundance output files per sample. Outputs were normalized to relative abundances and finally, merged into individual tables for all samples. The final output files were converted to biom-format for further analysis.

### Statistical Analysis

For analysis of DNA yield, proportion of host-derived, and final reads after quality control and host derived “contaminant” filtering, alpha diversity (using the Inverse Simpson index), a linear regression was performed where the predictors were sample type, extraction method, and an interaction term between sample type and extraction method to determine if the impact of extraction method on the final outcomes varied by sample type. We performed a secondary analysis on the stool samples which were collected between 3 and 18 years prior to determine the impact of sample storage on DNA yield. In this secondary analysis, we performed a linear regression where the predictors were duration of cryostorage (in years) and DNA extraction method.

For sequencing data, resulting sample by feature (either microbial taxonomy or predicted function) tables were merged with taxonomy tables and study covariates using the Bioconductor *phyloseq* package (McMurdie and Holmes, 2013) for downstream analyses. Taxa present in less than 5% of samples (in this case, taxa present in less than four samples), or predicted functional pathways in less than 10% of samples (in this case pathways present in less than eight samples) were excluded to filter out potentially spurious features due to sequencing or classification error (Callahan et al., 2016). We did not filter by relative abundance (i.e., rare taxa or pathways were retained if they were present in the threshold number of samples). Analyses were performed both on relative abundance and on centered-log-ratio (CLR) transformations; zero values were imputed prior to log-ratio transformation using the zCompositions R package (Palarea-Albaladejo and Martin-Fernandez, 2015). Measures of alpha diversity were calculated with the Inverse Simpson index. Measures of beta diversity were calculated using the Bray-Curtis dissimilarity index (Bray and Curtis, 1957) and the Aitchison distance (Aitchison et al., 2000). These two metrics were chosen as the former is commonly used in microbiome studies, and the latter accounts for the compositional nature of microbiome data (Gloor et al., 2017).

To test the hypothesis that extraction method predicted alpha diversity, we performed a mixed effects model where the predictors were sample type, extraction method, an interaction term between sample type and extraction method, adjusting for repeated measures in one subject. This was performed in all samples and also stratified by sample type (stool, sputum, dust). A secondary analysis was performed using Kruskall–Wallis testing because the measures of alpha diversity were not normally distributed, although this analysis does not account for repeated measures in a subject. To test the hypothesis that there were differences in microbial community composition or function based on extraction method, we performed permutational multivariate analysis of variance (PERMANOVA) (Anderson, 2001; McArdle and Anderson, 2001) with 10,000 permutations as implemented in the *vegan* R package (Oksanen et al., 2016). We first tested for an interaction between sample type and extraction method to determine whether the effect of extraction method on alpha or beta diversity differed by sample type; if the interaction term had a *p*-value of <0.20 (Selvin, 2004), we then performed subsequent PERMANOVA stratified by sample type.

To test the hypothesis that extraction method was associated with differential abundance of microbiota and microbial function, we first performed the appropriate transformation of the feature using the CLR approach as described above. In order to account for repeated measures (multiple aliquots of a single sample extracted by different methods), we performed boosted additive general linear models as implemented in the MaAsLin2 R package (Morgan et al., 2012). Features with a false discovery rate (FDR) of less than 10% were considered significant.

### Data Availability

The sequencing datasets for this study are uploaded to the NCBI Sequence Read Archive under SRA Accession Number PRJNA609351.

## Results

### DNA Yield

Treatment of samples differed only on DNA extraction method. Batch effects were controlled by randomizing all extracted DNA to one of two plates for library prep, and randomized again to one of two sequencing runs. DNA for all samples was eluted in an effective volume of 100 μL, with resulting DNA concentrations depicted in Table 1. When assessing the impact of extraction method on DNA concentration, we first performed a linear regression adjusting for extraction method, sample type, and an interaction term between extraction method and sample type. The interaction term between extraction method and sample type was statistically significant (*p* = 3 × 10^–6^) indicating that there was no single extraction method that had the highest DNA yield for all sample types. Promega had the highest DNA yield for sputum, whereas phenol:chloroform had the highest DNA yield for vacuumed dust and stool. Sample type was also a significant predictor for DNA yield (*p* = 3. 3 × 10^–11^), with vacuumed dust having the highest predicted DNA yield followed by stool and sputum. It should be noted that although vacuumed dust had the highest DNA yield, a larger proportion of the DNA is host-derived as compared to stool. Host-derived reads change resulting sequencing depth, as reads from human DNA are subsequently filtered out as part of the bioinformatics pipeline as described below.

**TABLE 1:** DNA yields stratified by sample type and extraction method.

Sample type	Extraction method	*N*	Mean ± SD	Median [IQR]
Dust	Phenol	6	56.5 ± 13.8	52.9 [49.2 – 63.3]
Dust	Promega	6	42.5 ± 6.6	40.7 [38.4 – 46.8]
Dust	Qiagen	6	46.7 ± 24.4	40.4 [35.5 – 56.8]
Dust	Zymo	6	0.6 ± 0.7	0.5 [0.1 – 0.9]
Sputum	Phenol	6	2.9 ± 2.8	2.6 [0.6 – 4.7]
Sputum	Promega	6	17.5 ± 13.8	13.3 [9.0 – 25.8]
Sputum	Qiagen	6	1.5 ± 2.0	0.7 [0.1 – 2.1]
Sputum	Zymo	6	3.3 ± 3.0	2.5 [1.6 – 3.5]
Stool	Phenol	6	42.4 ± 26.1	40.7 [23.9 – 61.6]
Stool	Promega	6	26.8 ± 6.6	25.5 [22.5 – 28.5]
Stool	Qiagen	6	10.5 ± 5.0	10.4 [9.3 – 12.7]
Stool	Zymo	6	3.1 ± 3.4	2.5 [0.3 – 5.1]
Mock	Phenol	1	26.4	26.4
Mock	Promega	1	14.4	14.4
Mock	Qiagen	1	12.0	12.0
Mock	Zymo	1	9.5	9.5
Reagent	Phenol	1	0.3	0.3
Reagent	Promega	1	−0.4	−0.4
Reagent	Qiagen	1	−0.4	−0.4
Reagent	Zymo	1	−0.4	−0.4

*Yields in ng/μL and presented as mean ± standard deviation (SD) or median [interquartile range (IQR)]. DNA quantified via a fluorometric method specific to double-stranded DNA. N, number of observations.*

In a secondary analysis examining the impact of sample storage on DNA yield, we focused on the stool samples which had been collected between 3 and 18 years prior to this study. Using linear regression, after adjusting for DNA extraction method, sample storage duration was a predictor of DNA yield, with each additional year of storage associated with a 1.0 ng/μL lower DNA concentration (*p* = 0.03).

### Total, Quality-Filtered, Host Sequencing Read

Across all sample types, there was an average of 22.6 million reads per sample. Several samples were not sequenced due to failed library prep, including one dust sample which underwent extraction by Zymo, two sputum samples (one which underwent extraction by phenol:chloroform, the other by Zymo), and all reagent-only negative controls with the exception of the phenol:chloroform negative control. Raw reads, host “contaminant” reads filtered out by mapping to a human genome reference database, and the final number of reads used as input for downstream taxonomic and functional analysis are depicted in Table 2, stratified by sample type and extraction method.

**TABLE 2:** Sequencing reads.

Sample type	Extraction method	*N*	Raw reads (Mean ± SD)	Host reads (Mean ± SD)	% Host reads (Mean ± SD)	Final reads (Mean ± SD)
Dust	Phenol	6	23,897,2743,117,506	11,848,9964,539,632	49.20%17.00%	9,098,9934,171,952
Dust	Promega	6	22,401,4742,189,300	10,164,0973,181,989	45.20%11.70%	9,399,2552,805,316
Dust	Qiagen	6	26,907,2994,204,592	11,724,1747,470,564	41.90%19.20%	11,779,7645,118,227
Dust	Zymo	5	20,844,5156,811,077	10,545,7294,067,631	52.40%16.70%	7,585,1084,903,690
Sputum	Phenol	5	23,438,3654,617,620	17,712,0355,468,234	74.90%12.10%	3,287,2942,683,193
Sputum	Promega	6	21,719,6243,302,827	16,394,7603,079,770	75.90%12.50%	3,045,2892,917,481
Sputum	Qiagen	6	19,935,7233,132,945	14,345,3536,086,910	69.60%20.40%	3,691,7293,183,922
Sputum	Zymo	5	19,672,4426,557,069	14,136,3006,061,229	73.00%20.80%	3,438,0664,070,224
Stool	Phenol	6	22,986,7136,263,653	40,93443,416	0.20%0.20%	21,027,9735,715,305
Stool	Promega	6	21,197,6522,217,871	101,331127,824	0.50%0.60%	19,261,6291,912,686
Stool	Qiagen	6	25,399,4663,734,027	39,36053,435	0.20%0.20%	23,342,5873,336,731
Stool	Zymo	6	18,088,2574,176,208	36,53444,855	0.20%0.30%	16,249,7754,145,873
Mock	Phenol	1	19,519,364	1,052	0.0%	17,183,276
Mock	Promega	1	20,236,264	1,490	0.0%	18,240,368
Mock	Qiagen	1	19,080,892	1,572	0.0%	17,303,262
Mock	Zymo	1	21,869,054	1,224	0.0%	20,010,718
Reagent	Phenol	1	9,024	341	3.8%	318
Reagent	Promega	0	NA	NA	NA	NA
Reagent	Qiagen	0	NA	NA	NA	NA
Reagent	Zymo	0	NA	NA	NA	NA

*Metagenomics shotgun sequencing was performed on extracted total gDNA using the Illumina HiSeq platform. Paired-end raw sequence reads in fastq format were processed further to quality trim the sequences, filter for Illumina phix sequences and trim the Illumina adapters using bbduk(3) (BBMap version 37.58). The trimmed fastqs were then mapped to a hg38 reference database (GCA_000001405.28, in this case human) using bowtie2 v.2.3.4.3 in order to remove host “contaminant” reads. Total number of raw reads, final reads (after quality trimming and filtering), number of mapped host reads, and % host reads (host reads divided by raw reads) by sample type and extraction method detailed below. Several samples failed library prep; one dust sample extracted with the Zymo approach, two sputum samples (one extracted with the phenol:chloroform approach and one with the Zymo approach), and three negative reagent controls extracted using the Promega, Qiagen, and Zymo approaches. Mean ± standard deviation of reads depicted. N, number of observations. SD, standard deviation.*

Extraction method was not a predictor for Either% host reads or the final number of filtered reads. However, the % host reads differed by sample type, with stool having on average only 0.3% host reads compared to dust (46.9% host reads) and sputum (73.3% host reads). Thus the final number of filtered reads is directly impacted by the % host reads (host reads are filtered out), with stool having the highest number of final reads and sputum the lowest number.

### Contamination in Negative Controls

Four reagent-only negative controls (one for each extraction method) was included in this study. Only the negative control from phenol:chloroform had detectable DNA (Table 2). All four negative controls underwent library prep; besides the phenol:chloroform reagent control, all other extraction controls failed library prep and were not sequenced. Analysis of the phenol:chloroform reagent control demonstrated a total of 9,024 sequencing reads, of which 318 passed quality filtering and host DNA “contaminant” removal. These reads were all derived from the *Escherichia* genus. For each sequencing run, elution buffer (either DEPC-Treated, DNase/RNase free molecular biology grade water or 25 mM Tris-HCl) was included in library preparation; all buffer-only samples failed library preparation and did not undergo sequencing.

### Bias Based on Mock Community Controls

**FIGURE 2:**
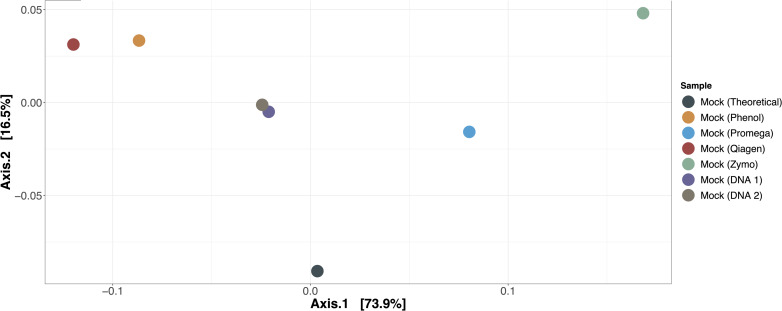
Ordination of mock communities compared to theoretical composition. Bray-Curtis dissimilarity was used to calculate differences in beta diversity between mock communities extracted by each method, the two mock community DNA samples used in each sequencing run, and the theoretical composition of the mock community. The mock community extracted by the Promega method most closely approximated the theoretical composition of the mock community.

The expected versus observed composition for mock microbial communities extracted by each method, as well as mock microbial community DNA included in each of the two sequencing runs, are depicted in Table 3. None of the samples exactly recapitulated the known composition of the mock community; this was also true for the mock microbial community DNA, suggesting that bias due to library preparation, sequencing, or our bioinformatics approach was present. However, when evaluating the mock microbial communities extracted by each method, Promega best recapitulated the composition of the mock community (Figure 2, ordination of mock communities using the Bray-Curtis dissimilarity index). The mock community extracted by the Promega approach was closest in distance to the theoretical composition of the mock community and the Zymo approach was the furthest [absolute Bray-Curtis distance to mock community as follows; Promega = 0.1066, mock DNA (sequencing run 2) = 0.1074, mock DNA (sequencing run 1) = 0.1201, phenol = 0.1545, Qiagen = 0.1733, Zymo = 0.2159]. Aitchison distance results were similar, demonstrating that the Promega approach most closely approximated the theoretical composition of the mock community. Given that we had only one replicate per mock community-extraction method combination, it was not possible to perform statistical analyses on the effect of extraction method on differential abundance in the mock communities. Qualitatively the phenol:chloroform and Qiagen approaches overrepresented *Lactobacillus fermentum*, whereas Zymo overrepresented *Escherichia coli* in the mock community samples.

**TABLE 3:** Mock community controls to identify bias in extraction method.

Species	Theoretical	Phenol	Promega	Qiagen	Zymo	Mock DNA 1	Mock DNA 2
*Pseudomonas aeruginosa*	12.00	8.81	14.39	8.02	15.07	16.16	15.25
*Escherichia coli*	12.00	10.43	15.50	9.84	20.55	12.19	11.76
*Escherichia* unclassified		0.40		0.49	0.60	2.03	1.24
*Salmonella enterica*	12.00	9.14	13.28	8.19	16.82	9.05	9.25
*Salmonella* unclassified		0.22	0.66	0.48	0.55	0.77	0.70
*Lactobacillus fermentum*	12.00	20.08	11.07	22.76	7.85	13.07	13.85
*Enterococcus faecalis*	12.00	15.01	14.83	13.71	16.01	11.43	12.14
*Staphylococcus aureus*	12.00	15.70	10.59	15.89	10.01	15.51	15.31
*Listeria monocytogenes*	12.00	10.85	8.30	11.40	6.29	10.49	10.81
*Listeria* unclassified						0.16	0.24
*Bacillus subtilis*	12.00	8.92	10.74	8.68	5.74	8.47	8.81
*Cryptococcus neoformans*	2.00	0.13	0.12	0.19	0.11	0.19	0.20
*Saccharomyces cerevisiae*	2.00	0.26	0.53	0.35	0.40	0.36	0.42
*Naumovozyma* unclassified			0.01		0.01	0.02	0.01
*Variovorax* unclassified						0.09	

*A mock community of microbes (Zymo D6300) with known composition as described in the “Theoretical” column was aliquoted in equal amounts, extracted according to each protocol, and underwent library prep for shotgun sequencing. In addition, mock community DNA (Zymo D6305) was also sequenced with each of two sequencing runs used in this study to identify possible bias resulting from library prep, sequencing, or our bioinformatics pipeline.*

### Assessment of Microbiota

**FIGURE 3:**
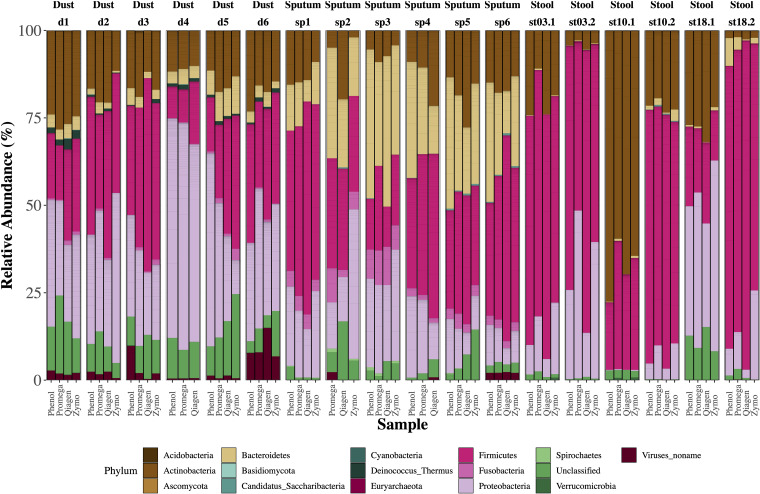
Relative abundance of microbes by sample type. Single bars represent one sample and each color shown represents one phylum. For each sample type (dust, sputum, stool), there were six biological replicates with four aliquots each which underwent a different extraction method. Several samples failed library prep including one dust sample (extracted by Zymo) and two sputum samples (one which underwent extraction by phenol:chloroform, the other by Zymo). For stool samples, the age of the sample is depicted with “stXX.” For example, “st03.2” refers to a stool sample stored for 3.2 years, “st10” refers to a stool sample stored for 10 years, and “st18” refers to a stool sample stored for 18 years.

**FIGURE 4:**
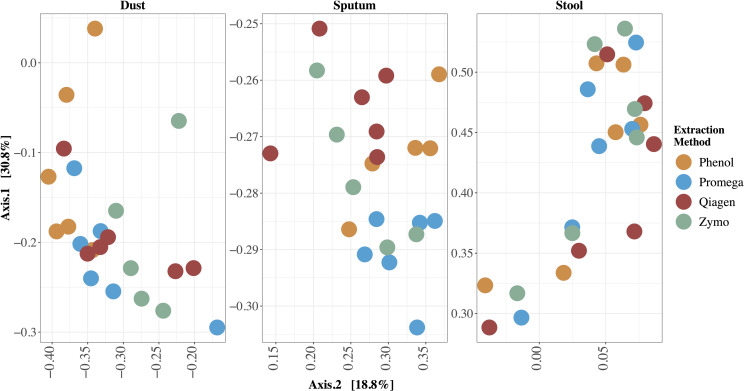
Principal Coordinates Analysis (PCoA) of differences in beta diversity between samples. Bray-Curtis dissimilarity was used to calculate beta diversity. Each dot represents one sample and each color represents one extraction method. Each sample is labeled with the sample identifier. In this type of visualization, samples with more similar microbial community structure cluster together whereas those with more dissimilar microbial community structure are a further distance apart. The effect of extraction method on resulting microbial community profiles differed by sample type; this effect was largest in dust and smallest in stool.

The relative abundance of microbes in each sample stratified by extraction method are depicted in Figure 3. We identified clades belonging to bacterial, archaeal, fungal, and viral kingdoms. The impact of extraction method on detected microbial community structure is shown in Figure 4, which depicts a Principal Coordinates Analysis (PCoA) based on Bray-Curtis dissimilarity. For statistical testing of these differences, we performed a permutational analysis of variance (PERMANOVA) where the covariates were subject (or location, in the case of the dust samples), in order to adjust for subject-specific effects, extraction method, sample type, and an interaction term between extraction method and sample type (Table 4). The interaction term was significant (*p* < 0.001, Bray-Curtis; *p* = 0.024, Aitchison) suggesting that the impact of extraction method on resulting microbial community structure differed across sample types. Therefore, we performed an analysis stratified by sample type to determine how the effects of extraction method on microbial community structure differed by sample type. In this stratified analysis, depending on the metric used (Bray-Curtis vs. Aitchison), extraction method accounted for between 3.0–3.9% of the variability in microbial community composition in stool samples, 9.2–12% of the variability in sputum samples, and 12–16% of the variability in dust samples.

**TABLE 4:** Permutational analysis of variance to determine influence of extraction method on microbial communities (both taxonomy and predicted function) stratified by sample type.

Outcome	Metric	Covariate	All samples		Stool only		Sputum only		Dust only	
			R2	*p*-value	R2	*p*-value	R2	*p*-value	R2	*p*-value
Taxonomy	Bray-Curtis	Extraction method	0.017	< 0.001	0.030	< 0.001	0.123	< 0.001	0.121	0.018
Taxonomy	Bray-Curtis	Sample type	0.477	< 0.001						
Taxonomy	Bray-Curtis	Extraction × Sample	0.023	< 0.001						
Taxonomy	Bray-Curtis	Subject	0.407	< 0.001	0.923	< 0.001	0.726	< 0.001	0.551	< 0.001
Taxonomy	Bray-Curtis	Residuals	0.076		0.047		0.151		0.328	
Taxonomy	Aitchison	Extraction method	0.021	0.002	0.039	0.003	0.092	0.026	0.156	0.002
Taxonomy	Aitchison	Sample type	0.47	< 0.001						
Taxonomy	Aitchison	Extraction × Sample	0.028	0.024						
Taxonomy	Aitchison	Subject	0.352	< 0.001	0.886	< 0.001	0.688	< 0.001	0.415	< 0.001
Taxonomy	Aitchison	Residuals	0.128		0.075		0.219		0.429	
Function	Bray-Curtis	Extraction method	0.011	0.003	0.066	0.002	0.108	0.031	0.226	< 0.001
Function	Bray-Curtis	Sample type	0.755	< 0.001						
Function	Bray-Curtis	Extraction × Sample	0.018	< 0.001						
Function	Bray-Curtis	Subject	0.179	< 0.001	0.864	< 0.001	0.715	< 0.001	0.557	< 0.001
Function	Bray-Curtis	Residuals	0.037		0.070		0.177		0.217	
Function	Aitchison	Extraction method	0.021	0.021	0.054	0.017	0.133	0.048	0.152	0.022
Function	Aitchison	Sample type	0.531	< 0.001						
Function	Aitchison	Extraction × Sample	0.032	0.055						
Function	Aitchison	Subject	0.268	< 0.001	0.844	< 0.001	0.454	< 0.001	0.398	< 0.001
Function	Aitchison	Residuals	0.148		0.102		0.412		0.449	

*Bray-Curtis dissimilarity index and the Aitchison distance results are presented.*

**FIGURE 5:**
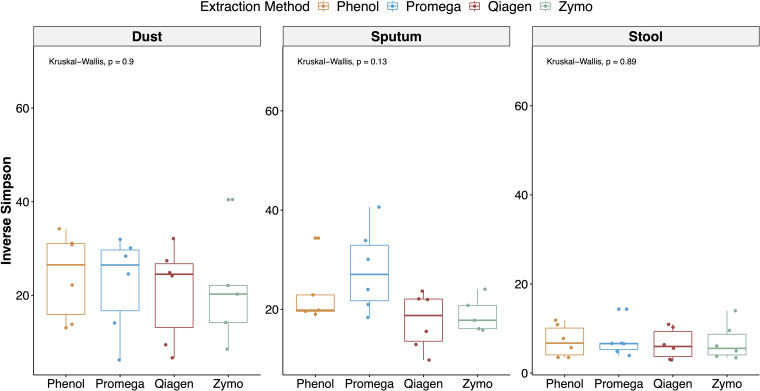
Alpha diversity stratified by sample type and extraction method. The inverse Simpson index (which accounts for both species richness and evenness) was calculated for each sample and aggregate results are depicted with boxplots, stratified by sample type and extraction method. In a linear regression adjusting for sample type, extraction method was not a significant predictor of alpha diversity.

Alpha diversity stratified by sample type and extraction method are depicted in Figure 5. Using a mixed effects model adjusting for repeated measures in a sample, extraction method was not a significant predictor of the Inverse Simpson Index (*p* = 0.060). However, in stratified analyses, extraction method was a predictor of alpha diversity in sputum samples (mixed effects model overall *p* = 0.0008), with the Promega and phenol:chloroform approaches having the highest alpha diversity in sputum samples (Promega vs. Zymo, *b* = 8.76, *p* = 0.001; phenol:chloroform vs. Zymo, *b* = 4.80, *p* = 0.067; Qiagen vs. Zymo, *b* = −1.55, *p* = 0.514). Using a Kruskal–Wallis test, extraction method was not a predictor of the Inverse Simpson Index (overall *p* = 0.580).

**FIGURE 6:**
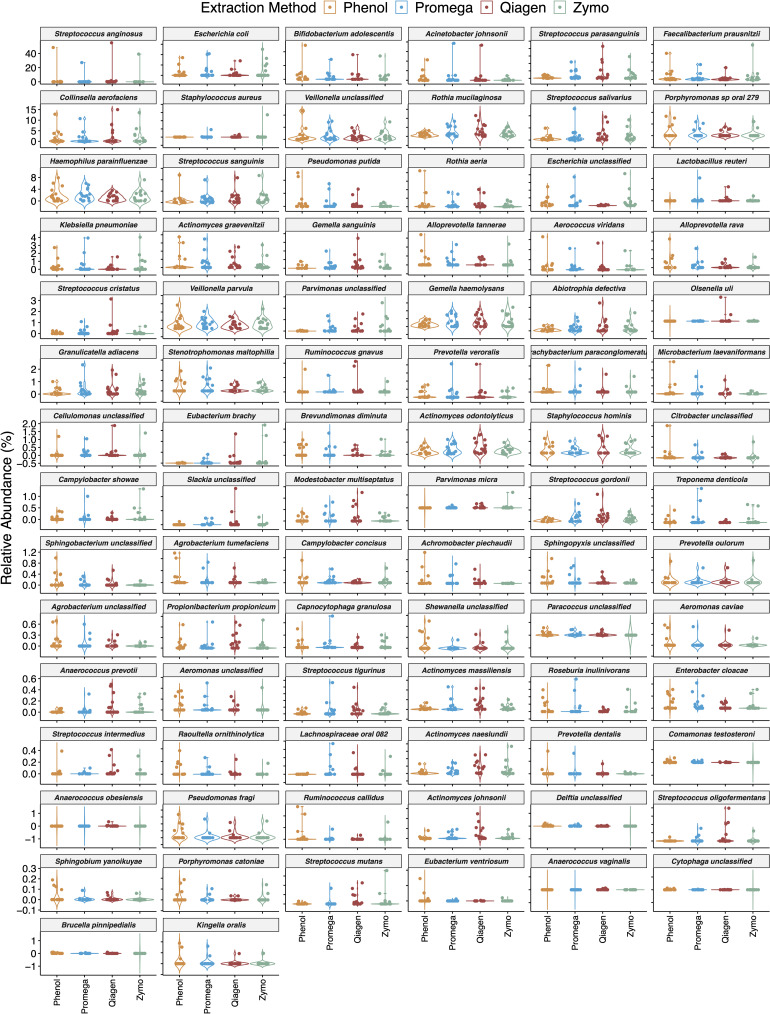
Impact of extraction method on differential abundance of microbiota. Boosted general linear models were performed on centered log-ratio transformed abundance and identified 86 microbial species with 113 pairwise comparisons that were differentially abundant based on extraction method. Here violin plots are sorted by decreasing relative abundance, and therefore the y-axis scale differs in each row.

The effect of extraction method on differential abundance in all samples is depicted in Figure 6 and Supplementary Table 1. Microbial species with an FDR < 10% in boosted linear models were considered significant. These models adjusted for the effect of sample type and repeated measures in a subject. In this analysis, 86 microbial species were found to be differentially abundant based on extraction method. These species include common environmental microbes such as *Pseudomonas putida*, human commensals such as *Streptococcus parasanguinis*, *Rothia mucilaginosa*, and *Lactobacillus reuteri*, and potential human pathogens such as *Escherichia coli*, *Staphylococcus aureus*, and *Serratia marcescens*.

### Assessment of Microbial Function

To determine the impact of extraction method on predicted microbial function detected by metagenomic sequencing, we performed permutational analysis of variance on both the Bray-Curtis dissimilarity index and the Aitchison distance to determine the impact of extraction method on overall patterns of predicted metabolic pathways (Table 4). The sample type^∗^extraction method interaction term was again statistically significant (*p* = 0.003, Bray-Curtis; *p* = 0.017, Aitchison) suggesting that the effect of extraction method on microbial function varied by sample type. In analyses stratified by sample type, extraction method accounted for 5.4–6.6% of the variability in stool, 11–13% of the variability in sputum, and 15–22.3% of the variability in dust.

The effect of extraction method on differential abundance of predicted microbial function in all samples is depicted in Supplementary Table 2. As with the taxonomic analysis, microbial species with an FDR < 10% in boosted linear models were considered significant, with models performing adjustment for both extraction method as well as sample type and repeated measures in a subject. One hundred and eleven different predicted metabolic pathways were differentially abundant based on extraction method, including pathways related to lipopolysaccharide biosynthesis, degradation of sugars and nucleic acids, and key metabolic functions such as oxygenic photosynthesis.

### Reagent Cost

We calculated the per-sample costs based on list prices for all reagents; the cost of laboratory consumables or labor was not included. These calculations may overestimate costs for some investigators who can access negotiated rates due to high volume use or through their institution. Estimates were calculated in U.S. dollars based on list prices as of October 17, 2019. The phenol:chloroform approach was the least expensive ($3.85 per sample), followed by Promega ($4.12 per sample) and Zymo ($5.41 per sample), with Qiagen being the costliest ($6.27 per sample). It should be noted that the phenol:chloroform method is the most labor-intensive, requiring more than twice the amount of hands-on time compared to the other methods.

## Discussion

In this study, we compare the impact of DNA extraction method on shotgun metagenomic profiles of diverse sample types including human stool, human induced sputum, and vacuumed dust. We find that the influence of extraction method on resulting microbial community structure and microbiome functional profiles differed by sample type, with differences in extraction method resulting in the least variability in stool and the most variability in vacuumed dust. Overall, in our hands and based on the methods tested, when considering our research priorities regarding DNA yield, success of metagenomic library prep and sequencing, absence of contamination noted in negative controls, the least amount of bias in positive mock community controls, and the ability to scale up extraction pipelines to a large number of samples, a protocol using the Promega Maxwell^®^ HT 96 gDNA Blood Isolation System had the best performance (Table 5). Our results highlight the importance of using a consistent protocol for DNA extraction for all sample types in a single study, and indicate that attempts to perform meta-analyses on sequenced microbiome data need to account for both extraction method and sample type as a confounder. This is particularly true for studies of non-fecal samples, where the impact of extraction method on resulting data is more pronounced.

**TABLE 5:** Summary of extraction methods tested.

Method	Key features	Reagent cost (per sample)	Advantages	Disadvantages	References*
Phenol:Chloroform	• Beadbeating • CTAB for lysis • Heat to 65°C	$3.85	• Does not require special equipment (Kingfisher Flex) • High DNA yield	• Contamination in negative controls • Labor intensive • Uses organic solvent requiring additional worker safety precautions	Fujimura et al., 2014; Levin et al., 2016; O’Connor et al., 2018; Levan et al., 2019; Rackaityte et al., 2020
Promega Maxwell HT 96 gDNA Blood Isolation (Technical Manual #TM473 per manufacturer)	• Beadbeating • CTAB for lysis • Heat to 95°C • Proteinase K	$4.12	• High-throughput • High DNA yield • Low bias	• Requires special equipment (Kingfisher Flex)	Petrof et al., 2013; Levin et al., 2016; Sinha et al., 2017; Chapagain et al., 2019; Clarke et al., 2019
Qiagen MagAttract PowerSoil DNA KF Kit (Earth Microbiome Protocol)	• Beadbeating • SDS for lysis • Heat to 65°C • RNase A	$6.27	• High-throughput	• Requires special equipment (Kingfisher Flex) • High cost	Easson and Thacker, 2014; McDonald et al., 2016; Halfvarson et al., 2017; Marotz et al., 2017; McDonald et al., 2018
ZymoBIOMICS 96 Magbead DNA Kit (Manufacturer instructions)	• Beadbeating • Proprietary lysing agent • Heat to 55°C • Proteinase K	$5.41	• High-throughput	• Requires special equipment (Kingfisher Flex) • Lower DNA yield • Some samples failed library prep	Terranova et al., 2018; Wurm et al., 2018; Zabat et al., 2018; Gibson et al., 2019; Reinhart et al., 2019

*This list is not exhaustive. Example references include five studies from a variety of journals, study types, and sample types.

Our findings extend the prior literature in several ways. First, one of our criteria used to evaluate each extraction method relied on shotgun metagenomics sequencing rather than amplicon sequencing; amplicon sequencing is the output currently used by the majority of the existing literature. As the cost of sequencing decreases, there is already a shift toward shotgun metagenomics sequencing for microbiome studies due to increased resolution of taxonomic and additional functional information. We show, for example, that while the effect of extraction method differs by sample type on both evaluation of taxonomy and function, the size of this effect modification was more pronounced when evaluating function rather than taxonomy for dust samples. Second, our study focused on diverse human and environmental sample types obtained from clinical studies conducted in different low- and high-resource settings; some of the stool samples used in this study had been stored at −80°C for over 18 years. While we show that duration of storage impacts DNA yield in stool samples, the effect size is small and should not limit the enthusiasm of investigators with archived samples for pursuing metagenomics studies. This finding is consistent with work by other investigators (Kia et al., 2016). Our data also highlights the unique challenges when working with non-fecal samples, including the larger proportion of human reads that effectively decrease sequencing depth and the lower biomass which accentuates signal from potential contamination. The problem of contamination in low biomass samples cannot be overstated (De Goffau et al., 2019; De Steenhuijsen Piters and Bogaert, 2020). In this study, we also quantify the proportion of human reads in sputum and dust samples, as this granularity may be useful for other groups planning microbiome studies using these sample types.

Our results are consistent with aspects of prior literature focused on DNA extraction methods. With our limited sample size, extraction method did not influence overall alpha diversity this finding is in agreement with another study that evaluated diverse human and environmental sample types with amplicon sequencing (Marotz et al., 2017), though different from studies focused on fecal samples (Claassen et al., 2013; Knudsen et al., 2016). These differences may be due to differences in beadbeating, a process that we standardized in this study to compare methods. A recent study evaluating the factors that contribute to well-to-well contamination found that this type of contamination primarily occurred during DNA extraction using plate-based methods [the Earth Microbiome Protocol (Marotz et al., 2017), Method 3 in our study] rather than “manual single-tube” extractions (Minich et al., 2019). Our findings show the opposite; only the manual single-tube approach had evidence of contamination whereas the three kit-based approaches (which were all plate-based) did not. This may have been due to several different factors. Our manual single-tube approach involved a phenol:chloroform protocol; this approach requires a large number of manual transfer steps from different tube types during the extraction process, whereas the single-tube spin-column approach has fewer steps. In addition, while we performed extractions on a 96-well plate (Methods 2 – 4), we performed beadbeating in single beadbeating tubes rather than 96-well beadbeating plates; this may have reduced the possibility of contamination. One limitation to our evaluation in this area is that we did not have multiple negative controls that would have increased the likelihood of detecting well-to-well contamination.

Strengths of our study include the diversity of sample types tested from existing and archived clinical studies conducted in multiple countries, the testing of high-throughput approaches to DNA extraction that may be applicable to large clinical studies, and the use of shotgun metagenomics sequencing to assess the performance of each extraction method. Our study does have limitations. We assessed a limited number of samples, and therefore it is possible that extraction method affects alpha diversity and we were not powered to detect these differences. We did not test a variety of bead-beating protocols as this would have greatly increased the number of samples for shotgun metagenomics sequencing which would have been too costly to perform. Our beadbeating protocol overall worked well for the sample types we tested in terms of detection of diverse types of microbes and success of library preparation. Beadbeating protocols are easily amenable to modification at no added cost, and can be fine-tuned in each investigator’s laboratory for the sample type of interest.

Overall, our findings show that high-throughput DNA extraction methods can perform well for a variety of sample types, even for samples archived for more than a decade. Beyond DNA yield, additional factors to consider when choosing DNA extraction method include presence of contamination and bias for easy-to-lyse organisms. Importantly, the influence of extraction method on microbiome profiles varies by sample type, and for some sample types may have a stronger influence on microbial functional profiles as compared to microbial community composition. Therefore, careful reporting of the DNA extraction method used in any one study and a consistent method across all samples is critical for interpretation of results. While removing bias completely is not possible with any DNA extraction method, use of positive controls such as a mock community or chemostat-manufactured samples can be used to quantify the direction of bias. Information provided by our study may assist investigators planning microbiome studies, especially those that integrate multiple sample types.

## Data Availability Statement

The datasets generated for this study can be found in the Sequence Read Archive; accession number PRJNA609351.

## Ethics Statement

The studies involving human participants were reviewed and approved by Research Ethics Committee of Mbarara University of Science and Technology, Ethical Review Committee of the icddr,b, Institutional Review Board of Massachusetts General Hospital/Partners Healthcare, Institutional Review Board of Boston Children’s Hospital. Written informed consent to participate in this study was provided by the participants or the participant’s legal guardian/next of kin.

## Author Contributions

PL, AW, and HS contributed to the design, analysis, interpretation of data, and drafting of the manuscript. AW, FQ, ER, WP, and PL provided samples for this study. All authors provided feedback and approval for the final submitted version of this manuscript.

## Conflict of Interest

The authors declare that the research was conducted in the absence of any commercial or financial relationships that could be construed as a potential conflict of interest.
