# Supplementary material for: Impact of DNA Extraction Method on Variation in Human and Built Environment Microbial Community and Functional Profiles Assessed by Shotgun Metagenomics Sequencing
Source: Front Microbiol. 2020 May 25;11:953. doi: 10.3389/fmicb.2020.00953 (PMC7262970; doi:10.3389/fmicb.2020.00953)
Supplement: Supplementary file 3 [file Data_Sheet_1.PDF]

Supplementary Material

1 Supplementary Table Overview

Supplementary Table 1. Effect of extraction method on differential abundance of microbiota. Boosted linear models on centered log-transformed abundance were performed to identify microbial species that were differentially abundant based on extraction method, adjusting for sample type and repeated measures in a subject. Microbial species with a false discovery rate (FDR) <10% were considered significant. 86 microbial species were differentially abundant by extraction method in 113 pairwise comparisons. Coefficients are listed based on individual pairwise comparisons, thus a microbial species may be listed more than once. Table arranged by increasing FDR.

Supplementary Table 2. Effect of extraction method on differential abundance of predicted microbial function. Boosted linear models on centered log-transformed abundance were performed to identify predicted microbial functions that were differentially abundant based on extraction method, adjusting for sample type and repeated measures in a subject. Microbial pathways with a false discovery rate (FDR) <10% were considered significant. 111 microbial species were differentially abundant by extraction method in 133 pairwise comparisons. Coefficients are listed based on individual pairwise comparisons, thus a microbial function may be listed more than once. Table arranged by increasing FDR.
